# Nanostructured TiO_2_ and PEDOT Electrodes with Photovoltaic Application

**DOI:** 10.3390/nano11010107

**Published:** 2021-01-04

**Authors:** Andrés Mauricio Ramírez, Linda Cattin, Jean-Christian Bernède, Fernando Raúl Díaz, Manuel Alejandro Gacitúa, María Angélica del Valle

**Affiliations:** 1 Laboratorio de Electroquímica y Materiales Aplicados, Centro de Nanotecnología Aplicada, Facultad de Ciencias, Universidad Mayor, Av. Alemania 0281, Temuco 4801043, Chile; andres.ramirez@umayor.cl; 2 Institut des Matériaux Jean Rouxel (IMN), CNRS, UMR 6502, Université de Nantes, 2 Rue de la Houssinière, BP 32229, 44322 Nantes CEDEX 3, France; linda.cattin-guenadez@univ-nantes.fr; 3 2MOLTECH-Anjou, CNRS, UMR 6200, Université de Nantes, 2 Rue de la Houssinière, BP 92208, 44000 Nantes, France; jean-christian.bernede@univ-nantes.fr; 4 Laboratorio de Electroquímica de Polímeros, P. Universidad Católica de Chile, Av. V. Mackenna 4860, Santiago 7820436, Chile; fdiaz@uc.cl; 5 Facultad de Química y Biología, Universidad de Santiago de Chile, Av. L.B. O’Higgins 3363, Santiago 7254758, Chile; manuel.gacitua@usach.cl

**Keywords:** dye-sensitized solar cell, electropolymerization, poly(3,4-ethylenedioxythiophene), polymer nanowires, solar cell, titanium dioxide

## Abstract

In this work, nanostructured TiO_2_ and poly-3,4-ethylenedioxythiophene (PEDOT) layers were electrochemically prepared over transparent electrodes. Morphological characterization evidenced the presence of nanostructures as planed with 50-nm-wide TiO_2_ rod formations followed by 30-nm-wide PEDOT wires. Different characterizations were made to the deposits, establishing their composition and optic properties of the deposits. Finally, photovoltaic cells were prepared using this modified electrode, proving that the presence of PEDOT nanowires in the cell achieves almost double the efficiency of its bulk analogue.

## 1. Introduction

Global warming is a current global concern known to be, in part, tightly linked to the large dependence of humans on non-renewable fossil-fuel-based energy sources involving the release of greenhouse gases. If renewable clean energy sources remain expensive, it will not be possible to reduce or stop global warming and its consequences. A valid strategy to lower the costs of clean-energy-harvesting devices, such as photovoltaic cells, could be to incorporate low-cost and more efficient materials, such as conducting polymers (CPs). This class of polymers has shown interesting electronic and optical properties, while maintaining feasible handling of plastics [1], with applications in supercapacitors, light-emitting diodes, electrochromic and photosensitive devices, sensors, batteries, transistors, fuel cells, electrocatalysis, and photovoltaics, among others [2,3,4,5,6,7,8,9,10,11,12,13,14,15,16,17].

CPs have proven their potential usefulness in energy-harvesting devices, and could be enhanced by a higher control of their morphology. A method using solely electrochemical perturbations has been developed ([18,19,20,21,22]; 10.1007/s11581-016-1796-9) as a solution for controlling the deposition process and electrode morphology, suitable for the reproducible construction of nanostructured semiconducting polymer layers. 

With respect to CP application in solar cells, most successful outcome corresponds to the incorporation of a doped poly-3,4-ethylenedioxythiophene (PEDOT) coating in a multiple-layer organic solar cell configuration. PEDOT is recognized as an excellent buffer layer material and some studies have revealed that it is possible to use it as an anode in a multiple-layer solar cell configuration [23]. Nevertheless, the performance of cells using this type of material, and other CPs, is limited by the imperfect connections (empty spaces) of a multiple-layer configuration. Short cuts, misguided excitons, and energy loss due to dissipative forces are some of the failure sources encountered when assembling these types of device [24,25]. A way to solve this problem is the incorporation of a nanostructured heterojunction assembly; nanostructured or nanowired (nw) assembly provides an enhanced surface-contact area while avoiding empty spaces between layers, and directs exciton movement correctly [26]. 

In previous studies, we have verified that PEDOT-nw displays better performance on other types of device, such as fuel cells [16]. Thus, it seems convenient to further test PEDOT-nw incorporation in the design of anodes for solar cells. In addition, some reports indicate that the previous addition of a semiconducting oxide layer as anatase (TiO_2_) followed by an appropriate sensitizer dye can enhance multiple-layer solar cell performance [27].

In the present study, transparent indium-doped tin oxide (ITO) electrodes are sequentially modified with layers of TiO_2_, sensitizer dye, mesoporous silica template, and nanowired PEDOT. Then, these electrodes are employed in the design of multiple-layer photovoltaic cells [28,29]. It is worth noting that most preparative methods are based on electrochemical techniques which ensures low cost and reproducible results. The main objective of the present report is to demonstrate that the electrochemical method enables sufficient control to produce a nanostructured conducting polymer layer over electrodes and to check if these deposits have advantages in terms of the performance of photovoltaic cells constructed with them [30].

## 2. Materials and Methods 

### 2.1. Electrode Modification

Electrochemical studies were carried out in a high-purity Ar atmosphere at room temperature (20 °C), in a three-compartment anchor cell, using a Pt wire with an area 20 times greater than that of the respective working electrodes. A reference wire of Ag|AgCl in tetramethylammonium chloride solution, with potential adjustment to that of the saturated calomel electrode (SCE) was used as the reference electrode [31]. The working electrode was glass coated with indium-doped tin oxide (ITO) of 0.21 cm^2^ geometric area and sheet resistance of ≤10 Ω sq^−1^.

ITO modification with TiO_2_ was done in a single compartment cell, from titanium oxysulfate 0.020 mol L^−1^ (TiOSO_4_ 99.9%, Aldrich, Santiago, Chile), as a precursor to titanium oxide; hydrogen peroxide 0.030 mol L^−1^ (H_2_O_2_ 30% *v*/*v*, Merck, Santiago, Chile) and potassium nitrate (KNO_3_, 99.0%, Merck), as a supporting electrolyte, in Milli-Q water, at pH 1.8 at 10 °C. The electro-obtaining of TiO_2_ was carried out by potentiostatic perturbance at −1.1 V for 300 s. Subsequently, the modified electrode was heat-treated at 500 °C for 1 h, following the heating ramp at 8 °C min^−1^. Then, the ITO|TiO_2_ electrodes were modified by immersing the electrode in a 1.0 × 10^−4^ mol L^−1^ N3 dye solution, for 24 h and then washed with acetone. N3 solutions corresponded to commercial cis-bis(isothiocyanate)-bis(2,2′-bipyridyl-4,4′-dicarboxylate)ruthenium(II) (C_26_H_16_N_6_O_8_RuS_2_, N3) in ethanol. Thus, ITO|TiO_2_|N3 electrodes were obtained.

**Figure 1:**
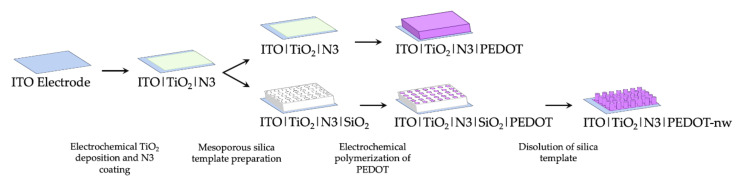
Schematic representation of preparative methods.

Incorporation of nanowired (PEDOT-nw) conducting polymer requires an early silica template preparation stage [10.1016/j.elecom.2020.106896] over the ITO|TiO_2_|N3 electrodes. Precursor solution for the silica template was prepared as follows: sodium nitrate 0.1 mol L^−1^ (NaNO_3_ 99.0%, Merck) as supporting electrolyte, tetraethyl orthosilicate 0.0034 mol L^−1^ ((C_2_H_5_O)_4_Si, 99.999%, Aldrich) as a precursor to silicon oxide and hexadecyltrimethylammonium bromide 0.115 mol L^−1^ as surfactant (CTAB, C_19_H_42_BrN, 95%, Aldrich), in ethanol 50% *v/v* (ethanol/Milli-Q = 1:1) under vigorous stirring for 2.5 h. Mesoporous silica template was prepared by immersing the ITO|TiO_2_|N3 electrodes in this solution and application of a potential step between −1.100 and −1.400 V. This modified electrode was washed with abundant Milli-Q water and dried in an oven for 24 h at 130 °C, to generate a thin film of SiO_2_. Next, the CTAB micelles were removed by immersing the electrodes for 15 min in acidified ethanolic solution under stirring. Then, the electrochemical growth of PEDOT nanowires took place inside the nano-channels of this template-modified electrode ([18,19,20,21,22]; 10.1007/s11581-016-1796-9). The electrochemical polymerization of 3,4-ethylenedioxythiophene (EDOT) was performed in a working solution containing monomer 0.010 mol L^−1^ (Aldrich) and support electrolyte 0.100 mol L^−1^ (tetrabutylammonium hexafluorophosphate, TBAPF_6_, Aldrich 99%), in acetonitrile (CH_3_CN 99.9%, Merck); the technique employed in this case was cyclic voltammetry (CV). Successive potentiodynamic scans were applied between −1.000 and 1.600 V on electrodes with and without the silica template for preparation of bulk and nanowired polymeric deposits. A scan rate of 0.1 V s^−1^ was used considering 5 and 1 potentiodynamic cycles for production of bulk PEDOT and PEDOT-nw, respectively. To remove the silica template and expose the nanowires, electrodes were immersed in 0.5 mol L^−1^ NaOH solution for 15 min, rinsed with 5% *m*/*v* NaHCO_3_ and finally with distilled water. Therefore, the ITO|TiO_2_|N3|PEDOT and ITO|TiO_2_|N3|PEDOT-nw electrodes were obtained. A schematic summary of the preparative methods can be seen in Figure 1.

### 2.2. Characterization

Surface morphology of the prepared electrodes was studied using scanning electron microscopy (SEM) Inspect F50 SEM or JOEL model 7600F (Nantes, France).

Identification of TiO_2_ deposit in its anatase form was confirmed by X-ray diffractometry (XRD) obtained on a Siemens D5000 spectrometer (Nantes, France) using Cu radiation (λKα 0.15406 nm) in an interval of 20 to 60 2θ and X-ray photoelectron spectroscopy using a Kratos Analytical AXIS Nova spectrometer, with an Al excitation line (Kα 14.866 eV) in an interval between 0 and 1.200 eV, analyzing the data with CasaXPS software (Nantes, France).

Electrochemical modifications and characterization of electrodes were made using a CH Instruments potentiostat/galvanostat controlled by CH750D software. UV-Vis spectra were recorded on a Specord 40 spectrophotometer (Analytik Jena, Thuringia, Germany), between 300 and 800 nm.

Solar cells were assembled following the methodology proposed by Bernéde et al. [29,30]. The photovoltaic characterization of the solar cells was carried out in an unautomated I–V under darkness and lighting, using a global solar simulator AM1.5 (Oriel 300 W, Nantes, France), with an intensity of 100 mW cm^−2^, which was adjusted with a reference photovoltaic cell (CIGS 0.5 cm^2^ PV, calibrated in NREL, Denver, CO, USA). 

## 3. Results and Discussion 

**Figure 2:**
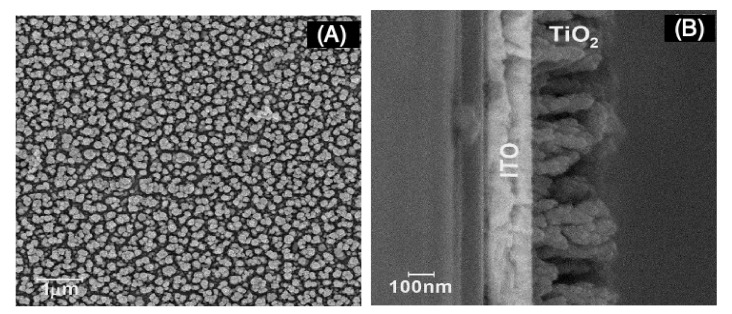
Scanning electron microscopy (SEM) micrographs of indium-doped tin oxide (ITO)|TiO_2_ after 300 s electrosynthesis time (**A**) top view and (**B**) cross section.

Before template and nanowire deposition, a layer of TiO_2_ in its anatase form was required to enhance the photocell performance. Morphological characterization is presented in Figure 2. 

Morphological characterization revealed rod-like formations of TiO_2_ over ITO surface after 300 s electrolysis time. From the pictures (Figure 2A,B), the rods were on average 50 and 300 nm in width and height, respectively, and rod density was calculated as being near 2.1 × 10^7^ per mm^2^. 

Other characterizations were considered. XRD characterization (Figure S1 in Supplementary Materials) showed expected results for the formation of anatase after 60 s deposition time displaying typical crystallographic signals [32], and the (101) phase became favored with electrolysis time. There was a small shift of TiO_2_ signals at the diffractograms, attributed in the literature to a strain resulting from planar stress very likely in thin-layer electrochemical deposits [33]. XPS analysis of the samples (Figure S2 in Supplementary Materials) showed signals of around 459.5 (Ti 2p^1/2^) and 465.5 (Ti 2p^3/2^) eV from the Ti–O interaction. 

**Figure 3:**
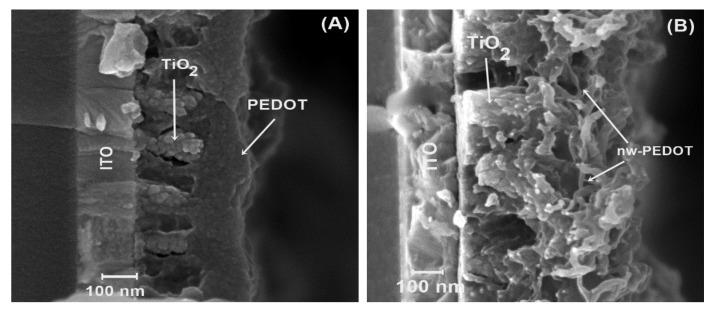
Electrodes’ morphological characterization. (**A**) ITO|TiO_2_|PEDOT and (**B**) ITO|TiO_2_|PEDOT-nw.

Afterwards, these electrodes were further modified with a dye to improve contact between TiO_2_ and the subsequent PEDOT layers. Bulk PEDOT and PEDOT nanowires were then electrochemically polymerized using a methodology published in past reports [16,17,18,20] (Figure 3). From the micrographs (Figure 3A,B), it can be seen that the polymer grows between the TiO_2_ structures with excellent cohesion for the hetero junction. Bulk PEDOT (Figure 3A) displays a homogeneous morphology, fills gaps between the TiO_2_ rods, and finishes with a smooth surface. On the other hand, nanostructured PEDOT, or PEDOT-nw (Figure 3B), displays nanometric wire morphology. PEDOT-nw shows wires with an average 30-nm width and, from micrograph analysis, a nanowire density of approximately 90,000 wires per μm^2^ can be calculated. 

To predict the behavior of the photocells, the energy diagrams of the respective modified electrodes, ITO|TiO_2_|PEDOT and ITO|TiO_2_|PEDOT-nw, were constructed (Figure S3 in Supplementary Materials) [34]. Electrochemical impedance spectroscopy measurements were made on electrodes in 0.1 mol L^−1^ LiCl solution, at 1 kHz to obtain Mott–Schottky graphs where flat band potential, Vfb, was calculated for TiO_2_, PEDOT and PEDOT-nw, finding values of −0.58, 0.674 and 0.113 V vs. SCE, respectively, which are consistent with those reported in the literature [35,36,37]. Thus, TiO_2_ showed a positive slope, which indicates the presence of an n-type semiconductor. On the other hand, PEDOT and PEDOT-nw negative slopes were found, accounting for a p-type semi-conductor. From these slopes, using Equations (1) and (2) [34], it is possible to calculate the apparent density of carriers, for the different semi-conductors, where *N*_D_ and *N*_A_ are the number of donors and acceptors for *n*-type and *p*-type semi-conductors, respectively.

(1) $$N_{D}=\frac{2}{\varepsilon \cdot \varepsilon_{0}\cdot e\cdot \;A^{2}\cdot P_{MS}}$$

(2) $$N_{A}=-\frac{2}{\varepsilon \cdot \varepsilon_{0}\cdot e\cdot \;A^{2}\cdot P_{MS}}$$

Here, *P*_MS_ is the value of the slope, *e* is the charge of the electron, ε_0_ and ε are the dielectric constant in vacuum and the dielectric constant of the semiconductor, considering values of 100 and 600,000 for TiO_2_ [38] and PEDOT [39], respectively, and A is the area. Therefore, the calculated values of 6.057 × 10^19^, 8.246 × 10^17^ and 1.107 × 10^17^ cm^−3^ are obtained for the respective TiO_2_, PEDOT and PEDOT-nw electrodes. These values are also consistent with those reported for these semiconductors [40,41,42]. Using the Tauc transformation [43], from the UV-Vis spectra (Figure S3, inserts at D,E,F in Supplementary Materials) the band gaps of the respective modified electrodes are determined, obtaining values of 3.65, 2.31 and 2.56 eV for TiO_2_, PEDOT and PEDOT-nw, respectively. The increase in the band gap of PEDOT-nw with respect to PEDOT is attributed or accounts for the quantum confinement of the nano-structured material. 

**Figure 4:**
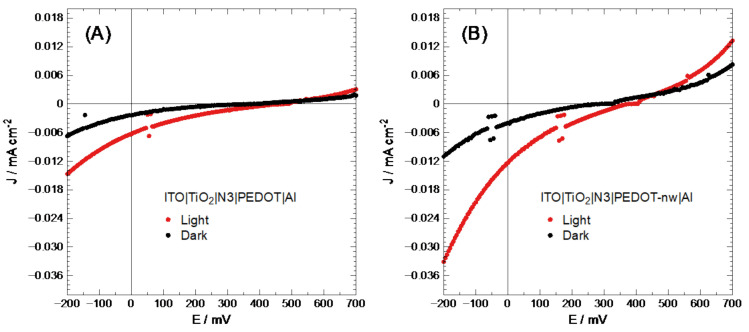
Solar cell J-V characterization of (**A**) ITO|TiO_2_|N3|PEDOT|Al and (**B**) ITO|TiO_2_|N3|PEDOT-nw|Al.

As has been proven in past reports [44], direct electro-synthesized conducting polymer deposit may show an imperfect connection between the semiconducting layers (short circuit). To improve cell performance, a layer of N3 dye was placed between the TiO_2_ and conducting polymer; this dye would also inject a significant amount of electron-hole pairs into the system, generating better efficiency as described in the literature [45]. The V values for each layer were transformed into eV in vacuum [46]. It should be noted here that organic semiconductors have flat band potential, corresponding to the bipolaron band, 0.70 eV above the HOMO level, unlike inorganic semiconductors, which have flat bands at 0.06 eV [34,47]. The energy level for N3 was taken from the literature [48], with −5.89 and −5.22 eV for HOMO and LUMO levels, respectively. With this information, band diagrams were constructed (Figure S4 in Supplementary Materials). These cells were then characterized based on their J-V performance, as shown in Figure 4.

At first sight, ITO|TiO_2_|N3|PEDOT-nw|Al electrodes show a higher current density than the modified ITO|TiO_2_|N3|PEDOT|Al electrodes. Table 1 summarizes the values corresponding to the photovoltaic characterization of these cells. 

The values in Table 1 show that nanowired PEDOT-based photovoltaic devices are practically twice as efficient as bulk polymer. Therefore, the selection of nanostructured presents advantages over bulk PEDOT deposits. Despite the fact that solar cell performance was not good, further studies will attempt optimizing through variation of nanowire length and/or diameter.

## 4. Conclusions

Electrochemical techniques are useful for the construction of nanostructured layers of photovoltaic cells such as TiO_2_ and PEDOT. The TiO_2_ layer was identified in its anatase form and showed a nanorod-ordered formation. Bulk and nanowired PEDOT deposits were successfully prepared over the initial TiO_2_ layer. In particular, PEDOT nanowire showed expected morphology and disposition as presented in past reports. 

To use these electrodes in photovoltaics, a layer of N3 dye was added between the TiO_2_ layer and the conducting polymer. The photocell architecture ITO|TiO_2_|N3|PEDOT-nw|Al showed the best current density–potential performance during characterization, meaning that the use of a nanostructured heterojunction in multiple-layer solar cells improves the overall performance.

## 5. Patents

The authors applied for an international patent called “Electrosynthesis of polymeric nanowires directly on solid surfaces (electrodes)”, with application numbers PCT/CL2018/050116 and reference 273176-WO.

## Figures and Tables

**Table 1:** Current-voltage (J-V) characterization photocells using bulk and nanowired PEDOT layers.

Deposited layer	V_OC_/V	J_SC_/mA cm^−2^	% FF	% η
PEDOT	0.48	6.0 × 10^−3^	16.1	4.9 × 10^−4^
PEDOT-nw	0.39	1.0 × 10^−2^	25.7	1.0 × 10^−3^

## Supplementary Materials

The following are available online at https://www.mdpi.com/2079-4991/11/1/107/s1, Figure S1: X-Ray diffractograms of ITO|TiO2 electrodes prepared by potential step of ࢤ1.100 during: (A) 60; (B) 180; (C) 300, and (D) 600 s; Figure S2: XPS analysis of ITO|TiO2 electrodes prepared by potential step of ࢤ1.100 V during 300 s: (A) Scan interval; (B) Maximum level of Ti 2p, and (C) Core level; Figure S3: Mott-Schottky graphics measured at 1 kHz: (A) TiO2; (B) PEDOT, and (C) PEDOT-nw obtained in absence of light. Graphics of (hν α L)2 vs. hν of: (D) TiO2; (E) PEDOT, and (F) PEDOT-nw; Figure S4: Energy diagram of (A) ITO|TiO2|N3|PEDOT and (B) ITO|TiO_2_|N3|PEDOT|PEDOT-nw.

## References

[1] Eftekhari A., Li L., Yang Y. (2017). Polyaniline supercapacitors. J. Power Sources.

[2] Bagheri H., Ayazi Z., Naderi M. (2013). Conductive polymer-based microextraction methods: A review. Anal. Chim. Acta.

[3] Abaci U., Guney H.Y., Kadiroglu U. (2013). Morphological and electrochemical properties of PPy, PAni bilayer films and enhanced stability of their electrochromic devices (PPy/PAni–PEDOT, PAni/PPy–PEDOT). Electrochim. Acta.

[4] Wang S., Kang Y., Wang L., Zhang H., Wang Y., Wang Y. (2013). Organic/inorganic hybrid sensors: A review. Sens. Actuators B Chem..

[5] Baker C.O., Huang X., Nelson W., Kaner R.B. (2017). Polyaniline nanofibers: Broadening applications for conducting polymers. Chem. Soc. Rev..

[6] Huang Y., Li H., Wang Z., Zhu M., Pei Z., Xue Q., Huang Y., Zhi C. (2016). Nanostructured Polypyrrole as a flexible electrode material of supercapacitor. Nano Energy.

[7] Salgado R., del Rio R., del Valle M.A., Armijo F. (2013). Selective electrochemical determination of dopamine, using a poly(3,4-ethylenedioxythiophene)/polydopamine hybrid film modified electrode. J. Electroanal. Chem..

[8] Zhao H., Yu S.H., Yoo P.J., Park J.H., Lee J.Y. (2013). Glucose Sensing by Glucose Oxidase/PEDOT Thin Film Electrode. Mol. Cryst. Liq. Cryst..

[9] Hernández L.A., del Valle M.A., Armijo F. (2016). Electrosynthesis and characterization of nanostructured polyquinone for use in detection and quantification of naturally occurring dsDNA. Biosens. Bioelectron..

[10] Friend R.H., Gymer R.W., Holmes A.B., Burroughes J.H., Marks R.N., Taliani C., Bradley D.D.C., Santos D.A.D., Brédas J.L., Lögdlund M. (1999). Electroluminescence in conjugated polymers. Nature.

[11] Nelson J. (2011). Polymer: Fullerene bulk heterojunction solar cells. Mater. Today.

[12] Mayer A.C., Scully S.R., Hardin B.E., Rowell M.W., McGehee M.D. (2007). Polymer-based solar cells. Mater. Today.

[13] Liu P., Yang H.X., Ai X.P., Li G.R., Gao X.P. (2012). A solar rechargeable battery based on polymeric charge storage electrodes. Electrochem. Commun..

[14] Bhattacharya R., de Kok M.M., Zhou J. (2009). Rechargeable electronic textile battery. Appl. Phys. Lett..

[15] Del Valle M.A., Gacitua M., Diaz F.R., Armijo F., Soto J.P. (2012). Electro-synthesis and characterization of polythiophene nano-wires/platinum nano-particles composite electrodes. Study of formic acid electro-catalytic oxidation. Electrochim. Acta.

[16] Del Valle M.A., Salgado R., Armijo F. (2014). PEDOT Nanowires and Platinum Nanoparticles Modified Electrodes to be Assayed in Formic Acid Electro-oxidation. Int. J. Electrochem. Sci..

[17] Ramírez M.R.A., del Valle M.A., Armijo F., Díaz F.R., Pardo M.A., Ortega E. (2017). Enhancement of electrodes modified by electrodeposited PEDOT-nanowires with dispersed Pt nanoparticles for formic acid electro-oxidation. J. Appl. Polym. Sci..

[18] Del Valle M.A., Gacitua M.A., Hernandez L.A., Díaz F.R. (2018). Electrosynthesis of Polymer Nanowires Directly onto Solid Surfaces (Electrodes).

[19] Del Valle M.A., Gacitúa M., Díaz F.R., Armijo F., del Río R. (2009). Electrosynthesis of polythiophene nanowires via mesoporous silica thin film templates. Electrochem. Commun..

[20] Del Valle M.A., Hernández L.A., Díaz F.R., Ramos A.C. (2015). Electrosynthesis and Characterization of Poly(3,4- ethylenedioxythiophene) Nanowires. Int. J. Electrochem. Sci..

[21] Del Valle M.A., Ramírez A.M., Hernández L.A., Armijo F., Díaz F.R., Arteaga G.C. (2016). Influence of the Supporting Electrolyte on the Electrochemical Polymerization of 3,4-Ethylenedioxythiophene. Effect on p- and n-Doping/Undoping, Conductivity and Morphology. Int. J. Electrochem. Sci..

[22] Ramírez A.M.R., Gacitúa M.A., Ortega E., Díaz F.R., del Valle M.A. (2019). Electrochemical in situ synthesis of polypyrrole nanowires. Electrochem. Commun..

[23] Rahman M.A., Rahim A., Maniruzzaman M., Yang K., Lee C., Nam H., Soh H., Lee J. (2011). ITO-free low-cost organic solar cells with highly conductive poly(3,4 ethylenedioxythiophene): P-toluene sulfonate anodes. Sol. Energy Mater. Sol. Cells.

[24] Krebs F.C. (2008). Degradation and stability of polymer and organic solar cells. Sol. Energy Mater. Sol. Cells.

[25] Jørgensen M., Norrman K., Krebs F.C. (2008). Stability/degradation of polymer solar cells. Sol. Energy Mater. Sol. Cells.

[26] Günes S., Neugebauer H., Sariciftci N.S. (2007). Conjugated Polymer-Based Organic Solar Cells. Chem. Rev..

[27] Karuppuchamy S., Nonomura K., Yoshida T., Sugiura T., Minoura H. (2002). Cathodic electrodeposition of oxide semiconductor thin films and their application to dye-sensitized solar cells. Solid State Ion..

[28] El Jouad Z., Barkat L., Stephant N., Cattin L., Hamzaoui N., Khelil A., Ghamnia M., Addou M., Morsli M., Béchu S. (2016). Ca/Alq3 hybrid cathode buffer layer for the optimization of organic solar cells based on a planar heterojunction. J. Phys. Chem. Solids.

[29] Barkat L., Hssein M., el Jouad Z., Cattin L., Louarn G., Stephant N., Khelil A., Ghamnia M., Addou M., Morsli M. (2017). Efficient hole-transporting layer MoO_3_:CuI deposited by co-evaporation in organic photovoltaic cells. Phys. Status Solidi A.

[30] El Jouad Z., Morsli M., Louarn G., Cattin L., Addou M., Bernède J.C. (2015). Improving the efficiency of subphthalocyanine based planar organic solar cells through the use of MoO_3_/CuI double anode buffer layer. Sol. Energy Mater. Sol. Cells.

[31] East G.A., Del Valle M.A. (2000). Easy-to-Make Ag/AgCl Reference Electrode. J. Chem. Educ..

[32] Natarajan C. (1996). Cathodic Electrodeposition of Nanocrystalline Titanium Dioxide Thin Films. J. Electrochem. Soc..

[33] Cullity B.D. (1956). Elements of X-ray Diffraction.

[34] Gelderman K., Lee L., Donne S.W. (2007). Flat-Band Potential of a Semiconductor: Using the Mott–Schottky Equation. J. Chem. Educ..

[35] Chettah H., Abdi D., Amardjia H., Haffar H. (2009). Electrosynthesis of TiO_2_ oxide film on ITO substrate and electrochemical comparative study of the oxide with its hydrated gel. Ionics.

[36] Boix P.P., Wienk M.M., Janssen R.A.J., Garcia-Belmonte G. (2011). Open-Circuit Voltage Limitation in Low-Bandgap Diketopyrrolopyrrole-Based Polymer Solar Cells Processed from Different Solvents. J. Phys. Chem. C.

[37] Gomes H.L., Taylor D.M. (1997). Schottky barrier diodes from semiconducting polymers. IEE Proc. Circuits Devices Syst..

[38] Kim S.K., Kim W.-D., Kim K.-M., Hwang C.S., Jeong J. (2004). High dielectric constant TiO_2_ thin films on a Ru electrode grown at 250 °C by atomic-layer deposition. Appl. Phys. Lett..

[39] Basavaraja C., Kim J.K., Huh D.S. (2015). Morphology and electrical properties of poly(3,4-ethylenedioxythiophene)/titanium dioxide nanocomposites. Macromol. Res..

[40] Wu J., Xu H., Yan W. (2016). Photoelectrocatalytic degradation Rhodamine B over highly ordered TiO2 nanotube arrays photoelectrode. Appl. Surf. Sci..

[41] Kirchartz T., Gong W., Hawks S.A., Agostinelli T., MacKenzie R.C.I., Yang Y., Nelson J. (2012). Sensitivity of the Mott–Schottky Analysis in Organic Solar Cells. J. Phys. Chem. C.

[42] Boix P.P., Garcia-Belmonte G., Muñecas U., Neophytou M., Waldauf C., Pacios R. (2009). Determination of gap defect states in organic bulk heterojunction solar cells from capacitance measurements. Appl. Phys. Lett..

[43] Tauc J. (1968). Optical properties and electronic structure of amorphous Ge and Si. Mater. Res. Bull..

[44] Brovelli F., Rivas B.L., Bernède J.C., del Valle M.A., Díaz F.R., Berredjem Y. (2007). Electrochemical and optical studies of 1,4-diaminoanthraquinone for solar cell applications. Polym. Bull..

[45] O’Regan B., Grätzel M. (1991). A low-cost, high-efficiency solar cell based on dye-sensitized colloidal TiO_2_ films. Nature.

[46] Trassatti S. (1986). The absolute electrode potential: An explanatory note. Pure Appl. Chem..

[47] Yang R., Smyrl W.H., Evans D.F., Hendrickson W.A. (1992). Evolution of polypyrrole band structure: A scanning tunneling spectroscopy study. J. Phys. Chem..

[48] Furube A., Murai M., Watanabe S., Hara K., Katoh R., Tachiya M. (2006). Near-IR transient absorption study on ultrafast electron-injection dynamics from a Ru-complex dye into nanocrystalline In2O3 thin films: Comparison with SnO_2_, ZnO, and TiO_2_ films. J. Photochem. Photobiol. A Chem..

