# Supplementary material for: Nanostructured TiO2 and PEDOT Electrodes with Photovoltaic Application
Source: Nanomaterials (Basel). 2021 Jan 4;11(1):107. doi: 10.3390/nano11010107 (PMC7824720; doi:10.3390/nano11010107)
Supplement: Supplementary file 1 [file nanomaterials-11-00107-s001.pdf]

Supplementary Materials: Nanostructured TiO_2 and PEDOT Electrodes with Photovoltaic Application

Andrés Mauricio Ramírez [1], Linda Cattin [2], Jean-Christian Bernède [3], Fernando Raúl Díaz [4], Manuel Alejandro Gacitúa [5] and María Angélica del Valle [4,*]

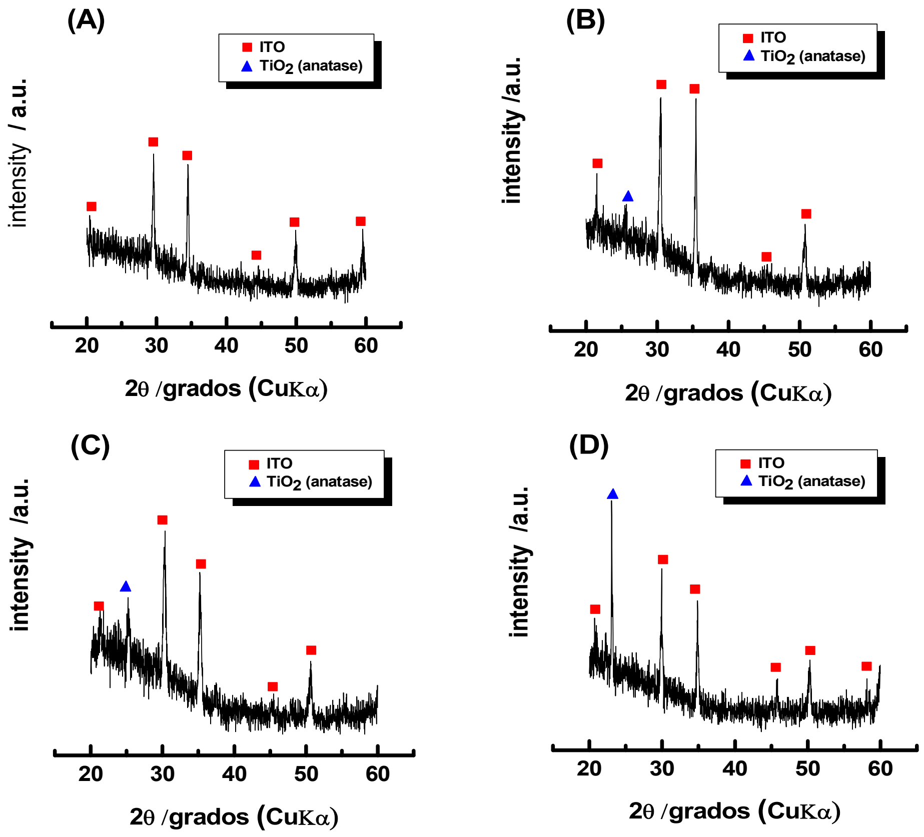

Figure S1. X-Ray diffractograms of ITO|TiO_2 electrodes prepared by potential step of −1.100 V during: (**A**) 60; (**B**) 180; (**C**) 300, and (**D**) 600 s.

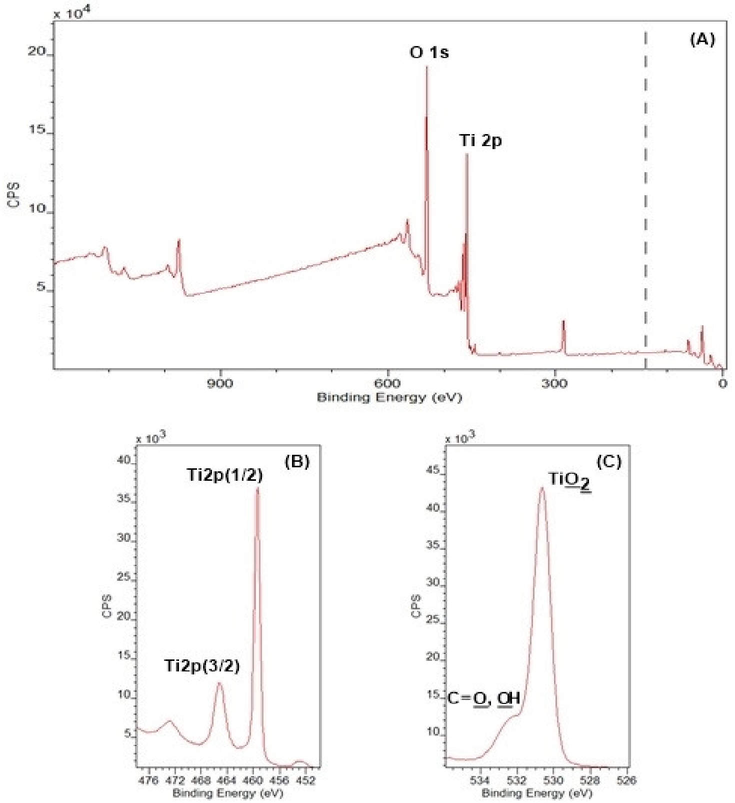

Figure S2. XPS analysis of ITO|TiO_2 electrodes prepared by potential step of -1.100 V during 300 s: (**A**) Scan interval; (**B**) Maximum level of Ti 2p, and (**C**) Core level.

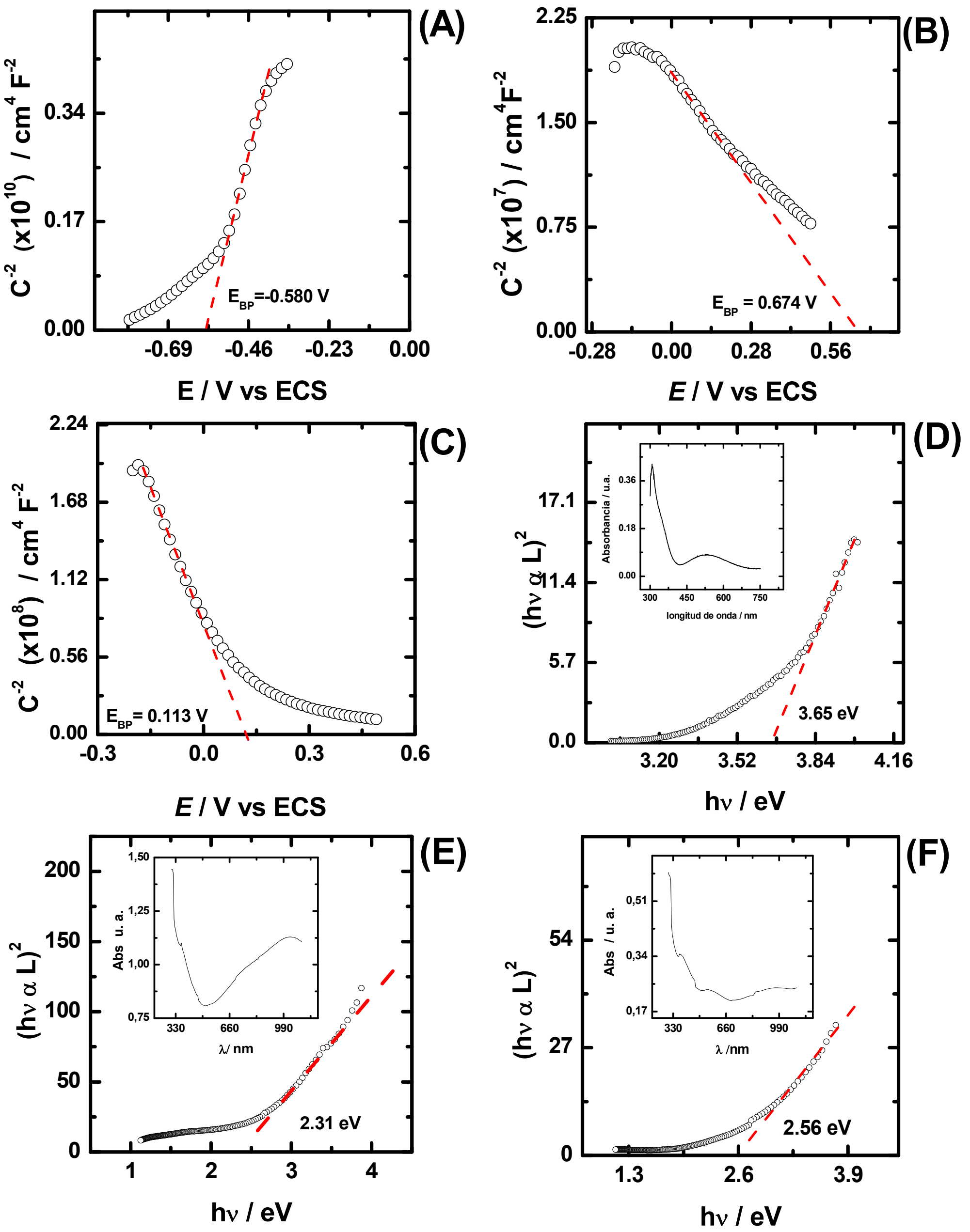

Figure S3. Mott-Schottky graphics measured at 1 kHz: (**A**) TiO_2; (**B**) PEDOT, and (**C**) PEDOT-nw obtained in absence of light. Graphics of $(h\nu\ \alpha\ L)^2$ vs. $h\nu$ of: (**D**) TiO_2; (**E**) PEDOT, and (**F**) PEDOT-nw.

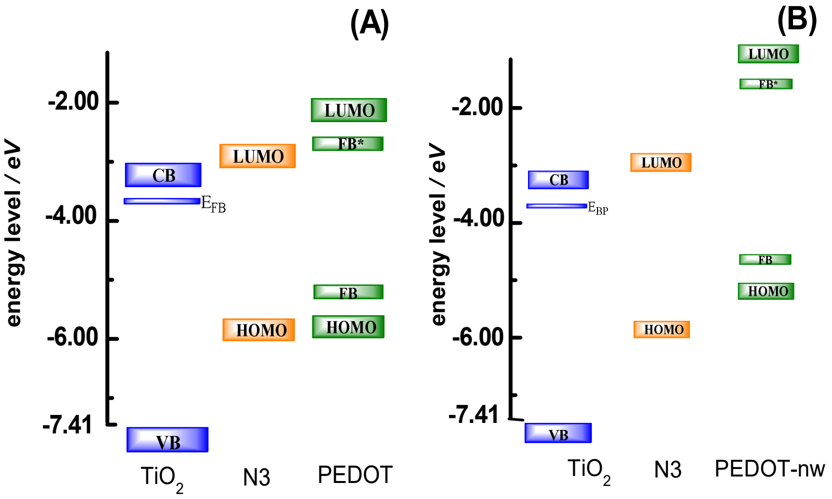

Figure S4. Energy diagram of (**A**) ITO|TiO_2|N3|PEDOT and (**B**) ITO|TiO_2|N3|PEDOT|PEDOT-nw.
